# Chromosome-level *de novo* genome assembly of wild, anoxia-tolerant crucian carp, *Carassius carassius*

**DOI:** 10.1038/s41597-025-04813-3

**Published:** 2025-03-24

**Authors:** Laura Marian Valencia-Pesqueira, Siv Nam Khang Hoff, Ole K. Tørresen, Sissel Jentoft, Sjannie Lefevre

**Affiliations:** 1 Section for Physiology and Cell Biology, Department of Biosciences, University of Oslo, Oslo, Norway; 2 Centre for Ecological and Evolutionary Synthesis, Department of Biosciences, University of Oslo, Oslo, Norway

**Keywords:** Animal physiology, Genome, Genomics

## Abstract

Crucian carp (*Carassius carassius*), a member of the carp family (Cyprinidae), is known for its remarkable anoxia tolerance. The physiological responses and adaptations to anoxia are well documented, but there is a need for better understanding of the molecular regulation and evolutionary mechanisms behind these adaptations. Here we present a high-quality, functionally annotated, chromosome-level genome assembly that can facilitate such further studies. Genomic DNA was obtained from a wild-caught crucian carp specimen and used for PacBio long-read, Illumina short-read and Hi-C sequencing. Short-read mRNA data were used for structural annotation using the BRAKER3 pipeline, while PacBio long-read RNA sequencing data were used for annotation of untranslated regions and refinement of gene-isoform relationships, using the PASA pipeline. The full assembly had a contig-level N50 of 15Mbp in 290 scaffolds and 98.6% of the total length (1.65Gbp) placed in 50 chromosomes. Structural annotation resulted in 82,557 protein-coding transcripts (in 45,667 genes), with a BUSCO completeness of 99.6% and of which 77,370 matched a protein in the UniProtKB/Swiss-Prot database.

## Background & Summary

Crucian carp (*Carassius carassius*) is a wide-spread species in Northern Europe, normally found in smaller ponds and lakes with rather harsh environmental conditions. In some ponds crucian carp may even be the only fish species present. Due to the small surface area and little or no current in some ponds, ice forms during the winter and prevents oxygen from diffusing into the water from the air. When the layer of ice becomes covered with snow, UV radiation from the sun is effectively blocked, preventing photosynthesis and thus replenishment of the oxygen that continues to be used by all remaining organisms. Consequently, the ponds eventually become depleted of oxygen (anoxic) until the ice melts in the spring. Contrary to most other vertebrates^1^, the crucian carp can survive anoxia for months, explaining why it is often the sole fish species in ponds with seasonal anoxia. The physiological adaptations allowing it to survive anoxia are fairly well characterized^2,3^, with one key trait being the ability to convert the anaerobic end product lactate into ethanol, which can be excreted to the water via the gills, contrary to lactate that would accumulate in tissues and lead to severe acidosis. It has been shown that the pyruvate dehydrogenase complex of crucian carp has an additional and modified subunit of the E1 enzyme^4^, which is highly expressed in muscle tissue during anoxia and thought to have pyruvate decarboxylase activity, i.e. converting pyruvate into acetaldehyde, which can then be converted into ethanol by alcohol dehydrogenase. The carp-specific whole-genome duplication^5–7^ has been hypothesized to play a central role in the development of anoxia tolerance by enabling neofunctionalization of gene paralogs such as the extra E1 subunit^4^.

Here, we present a high-quality reference genome (74x coverage), that has been scaffolded using chromosome conformation capture (Hi-C) sequencing, and structurally and functionally annotated based on transcriptomic evidence. This genome assembly will open opportunities to study the molecular and evolutionary basis of anoxia tolerance in the crucian carp. The genome will also be useful for furthering research on evolutionary and genomic aspects of fish species that have undergone genome duplications. Interestingly, the anoxia tolerance of the closely related goldfish (*Carassius auratus*) is markedly lower than that of crucian carp (i.e. shorter survival time^8^), and anoxia tolerance in silver crucian carp (*Carassius gibelio*) has to our knowledge not been reported. Similarly, the common carp (*Cyprinus carpio*) from the same family of fish that underwent the carp-specific whole-genome duplication^6^ is only somewhat hypoxia tolerant^9^ and not anoxia tolerant. A specific comparison of the genes involved in known physiological and metabolic functions of anoxia tolerance, between crucian carp, silver crucian carp, goldfish, and common carp, can be the first step to shed light on what is still present in the anoxia-tolerant fish, and what was lost in the less tolerant fish. A comparison with the existing genomes of a farmed-type crucian carp^10^, silver crucian carp^11^, goldfish^12^, and common carp^13^, indicate that the genome we present here is more contiguous and more complete. This genome thus represents a necessary contribution to the larger effort of investigating anoxia tolerance from the genomic and transcriptomic point of view and elucidating the evolutionary history of the physiological mechanisms. Having a high-quality genome of a crucian carp specimen from a population known to be recurrently exposed to anoxia^14^ (such as Tjernsrudtjernet in Oslo, Norway) and with an extensively characterised physiology and response to anoxia^2–4,15–23^, will be valuable for future studies linking physiological adaptations to molecular regulation and evolution. The genome will also be useful for studies of population genomics. Crucian carp from different ponds in Norway have been shown to have different morphology^24^ related to the presence or absence of predators, and population genomics can be used to investigate the genomic basis of these differences. It would also be useful to compare with other populations in Northern Europe (e.g. the farmed UK populations and wild populations in Finland), where the habitats may vary with regards to the extent of seasonal anoxia. In summary, the high-quality genome presented here will be an important resource for the field of comparative animal physiology, and fish ecology and evolution.

## Methods

### Sample acquisition

#### Specimens

The male crucian carp specimen selected for whole genome sequencing stems from a batch of crucian carp collected from a small pond in Oslo (‘Tjernsrudtjernet’; N 59.922886 E 10.609834) using nylon net cages. The fish were captured in October 2019 and held at 10–12 °C in the InVivo Aquarium facility (Depart. Biosciences, Univer. Oslo) for approximately three months. The fish were fed by hand to satiation with commercial carp pellets twice daily (Tetrapond, Tetra, Melle, Germany), and kept under a 12 h:12 h light-dark cycle in 750 L tanks with a semi-closed recirculation system of aerated and dechlorinated tap water. At the time of sampling (in January 2020), the selected specimen was euthanized with a sharp blow to the head, after which blood was sampled by caudal puncture. A portion of the blood was preserved in ethanol while the remaining portion was flash frozen using liquid nitrogen, as were remaining tissues (brain, liver, red muscle, white muscle, gills, gonad, spleen, kidney, heart).

For structural annotation (using short-read RNA sequencing) samples were taken from multiple individuals exposed to normoxia and from different tissues. This collection included samples from kidney, spleen, gills, gonad (male and female), skin, scales, intestine, eye, liver, red muscle, and white muscle (from a batch collected 12 Oct 2021 from the same pond as mentioned above and sampled 11 Aug 2022). Additionally, brain tissues were sampled from a batch collected 13 September 2013 and sampled 19 November 2014 and heart were sampled from a batch collected 23 September 2022 and sampled 8 June 2023. The fish were given minimum 2 weeks to acclimatize to holding conditions prior to any experiment or sampling. Individuals of both sexes were included to increase genetic diversity of the transcriptomic data. For the brain, samples were from three individuals, exposed to 6 days normoxia, 6 days anoxia, or 6 days anoxia followed by 1 day re-oxygenation, respectively. For the heart, one sample was from a fish exposed to normoxia for 1 day and another sample was from a fish exposed to anoxia for 2 days. Tissues were flash frozen on liquid nitrogen and stored at −80 °C. The anoxia-exposure experiments were carried out according to Norwegian animal research guidelines (‘Forskrift om bruk av dyr i forsøk’) at the InVivo Aquarium facility approved by the Norwegian Food Safety Authorities (approval no. 155/2008).

#### DNA and RNA extraction

For preparation of long-read and short-read DNA libraries, genomic DNA was extracted from 25 mg of blotted dry-weight muscle tissue using the Circulomics Nanobind HMW Tissue DNA kit (Handbook v06.16 3/2019), to obtain 263 ng/µL of DNA with modal peak size distribution of 47 kb. This DNA was used for the library preparation of PacBio long reads (for genome assembly) and Illumina short reads (for error correction of the genome assembly). For preparation of the Hi-C library (cross-linked DNA in close proximity for chromosome conformation capture), genomic DNA was extracted from blood in ethanol using the Arima-HiC kit with a modified version of the mammalian blood protocol. Specifically, the sample was washed with PBS and the ethanol removed, and then continued from step 12 in the Arima blood protocol.

The RNA for both short- and long-read sequencing was extracted using the TRIzol reagent (Cat. no. 15596026 and 15596018), following instructions from the manufacturer. The extracted RNA from different tissues was pooled (except the brain samples that were processed previously) and checked for integrity using a Bioanalyzer.

### Library preparation and sequencing

The library preparation and sequencing were provided by the Norwegian Sequencing Centre (www.sequencing.uio.no), a national technology platform hosted by the University of Oslo.

#### Long-read and short-read DNA sequencing

The long-read DNA library was prepared using the Pacific Biosciences Express library preparation protocol without any fragmentation of the sample prior to library preparation. Size selection of the final library was performed using BluePippin with a 15 kb cut-off. The long-read library was sequenced on one 8 M SMRT cell on the Sequel II instrument using Sequel II Binding kit 2.0 and Sequencing chemistry v2.0. Loading was performed by diffusion (movie time: 15 hours). The sequencing yielded 5.8 M reads with an N50 insert length of 23 kb. The short-read DNA library was built from 1000 ng of genomic DNA using the Kapa Hyper prep PCR free workflow. For quality check, the library was amplified with PCR, purified, and checked with Fragment Analyzer and NGS kit. The library was sequenced on one lane Illumina HiSeq 4000 with 300 cycles (150 bp reads, paired end), yielding 297 M read pairs.

#### Hi-C sequencing

A quality control of the genomic DNA (following the Arima protocol) confirmed that the sample included correctly cross-linked proximal DNA, and therefore was ready for library preparation using the Arima library protocol. First, 3.4 µg of cross-linked DNA sample (from Arima kit) were sheared in Covaris tubes and Covaris E220 instrument. Then, after size selection, the biotin enrichment step used 382 ng of sheared DNA, followed by ligation using Illumina unique adaptors. The library was amplified with 10 cycles of PCR, checked in fragment analyzer (FA) and NGS kit. Finally, the Kapa Quantification kit was used for assessment of library concentration. The library was sequenced on one lane Illumina HiSeq 4000 with 300 cycles (150 bp read paired end), yielding 343 M read pairs.

#### Long-read and short-read RNA sequencing

The long-read RNA libraries were prepared from total RNA from each tissue using Pacific Biosciences protocol for Iso-Seq™ Express Template Preparation for Sequel® and Sequel II Systems. The libraries were multiplexed and sequenced on the PacBio Sequel II instrument using one SMRT cell with Sequel II Binding kit 2.0 and Sequencing chemistry v2.0 (loading by diffusion) and yielding 5,860,324 subreads with an average subread length of 3,093 bp. IsoSeq analysis to obtain full-length transcripts from subreads was performed using the IsoSeq pipeline (SMRT Link v9.0) with default parameters. Reads were demultiplexed prior to filtering for full-length reads and clustering of isoforms. This processing resulted in 223,902 high-quality isoforms. Polished ccs reads (3,137,504) were later created from the raw subreads using the PacBio command-line tool ‘ccs’ (SMRT Tools v10.1) with default filtering parameters.

RNA-samples from diverse tissues of the crucian carp were pooled into 5 different sets to be sequenced as independent libraries to ensure sufficient read coverage from all sets. Each set was prepared with Strand-specific TrueSeq^TM^ mRNA-seq library prep and all the sets were sequenced together in one ¼ S4 Illumina Novaseq 6000 flow cell. Heart samples from other fish in normoxia and anoxia were included in the same sequencing run. Additionally, already available brain RNA-seq data from a previous project was included (strand-specific TruSeq mRNA libraries multiplexed on 4 lanes Illumina HiSeq 2500; 250 cycles, paired end).

### Genome assembly and annotation

**Fig. 1:**
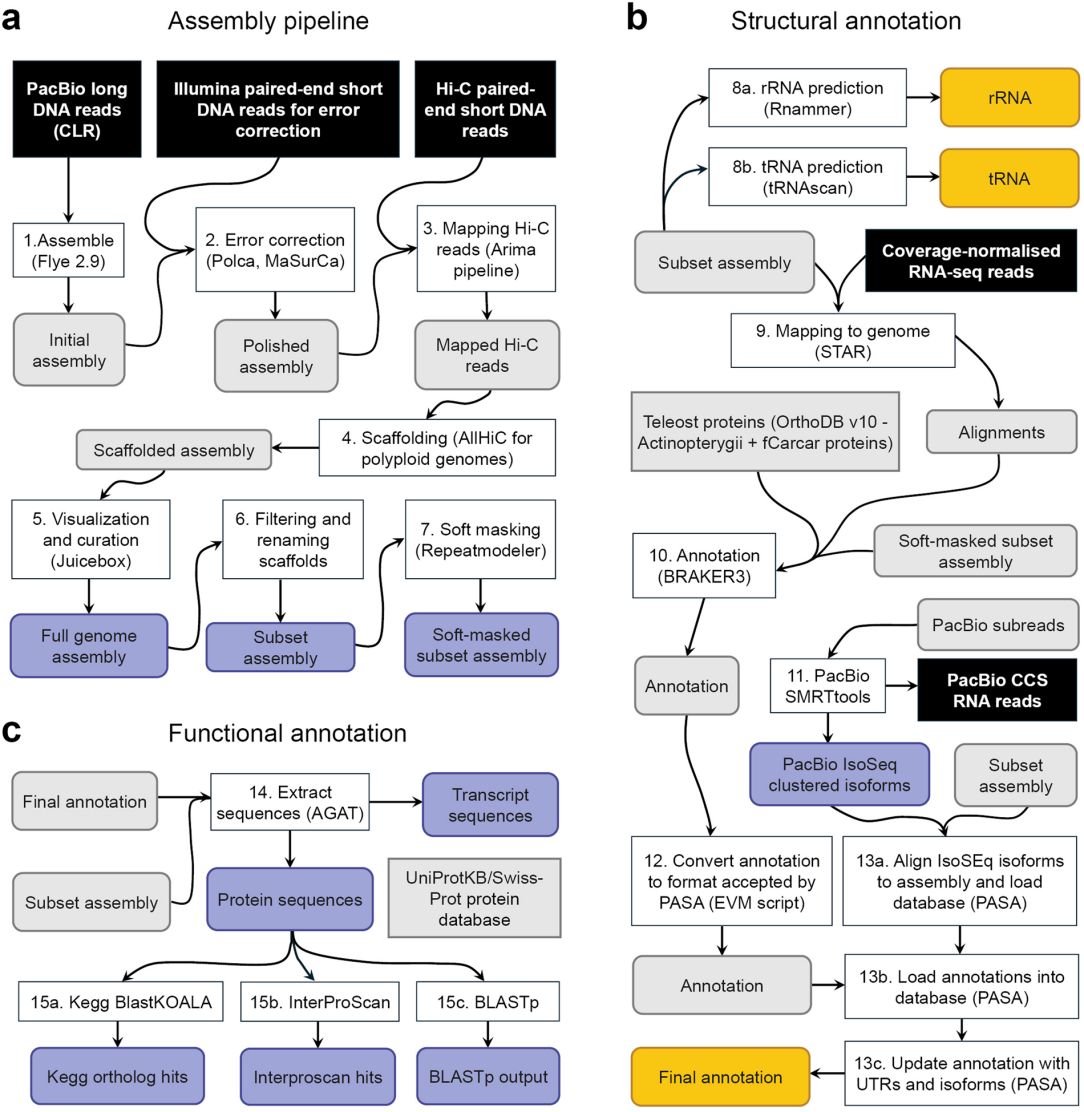
Overview of bioinformatic assembly and annotation pipelines. (**a**) *De novo* assembly of PacBio long DNA reads (1) followed by error correction using 150 bp Illumina DNA reads (2), mapping and scaffolding with proximity-ligated (Hi-C) DNA reads (3,4), visualization of contact maps and manual curation (5), resulting in a fully assembled genome with 290 scaffolds. Sequences were renamed and contigs > 2995 bp were kept, resulting in a final subset genome with 262 scaffolds (6) and a soft-masked version (7). (**b**) Ribosomal RNA, rRNAs (8a) and transfer RNAs, tRNAs (8b) were annotated from the genomic sequences, while models of protein coding genes were predicted from mapped RNA-seq reads (9) and a protein dataset consisting of the OrthoDB v10 ‘Actinopterygii’ dataset plus predicted proteins from the farmed UK crucian carp genome (fCarCar2; GCF_963082965.1) (10). Protein-coding gene models were further revised to improve UTR annotation and gene-to-isoform relationships using full-length transcript (PacBio IsoSeq clustered isoforms) (13), resulting in a final set of protein-coding gene models (12). (**c**) Functional annotation was carried out by extracting transcript and protein sequences (14) and searching for the protein sequences in different databases (15). Details of software packages and scripts used, including versions, are provided in Table 1, while details of input and final output files are provided in Table 2.

Table 1 lists all the software and versions used in our pipelines, as described in more detail below. A schematic overview of the assembly and annotation steps is provided in Fig. 1. Unless otherwise indicated, computations were carried out on a high-performance computing cluster.

**Table 1:** Software packages and pipelines used for assembly and annotation.

Step	Description	Software	Version
Assembly	Genome assembly	Flye	2.9
	Error correction	POLCA (MaSuRCA)	4.0.1
	Aligner for POLCA	BWA	0.7.17
	Adapter trimming	TrimGalore	0.6.6
	Mitochondrial genome	Mitofish (MitoAnnotator)	3.87
Scaffolding	Mapping of Hi-C data	Arima pipeline	1
	Arima pipeline dependencies	BWA	0.7.17
		Picard	2.22.1
		SAMtools	1.1
	Scaffolding with Hi-C data for genomes with high ploidy	AllHiC pipeline	0.9.8
	Tools for Hi-C data	Matlock	1
	Matlock dependencies	HTSlib	1.9
		GSL	2.5
		ngsLD	191108
		Java	11.0.2
	Hi-C map visualization and genome curation	Juicebox	1.11.08
		Python	3.7.4
Assembly quality	Genome contamination check	FCS-GX	0.3.0
	Calculation of optimal k-mer size	Kmergenie	1.7051-7
	K-mer quality control	Genomescope	2017
	K-mer counts	Jellyfish	2.3.0
	‘snail plot’ for quality overview	Blobtoolkit	4.2.1
	Continuity metrics	QUAST	5.0.2
	Completeness validation with actinopterygii_odb10	BUSCO	5.4.7
Annotation	Mapping reads to genome	STAR	2.7.11a
	Soft-masking	RepeatModeler2	2.0.1
	Primary structural annotation	BRAKER3	3.0.7.5*
	Convert braker3.gtf to evm.gff3	EVidenceModeler	2.1.0
	UTR and isoforms annotation	PASA	2.5.3*
	Search against multiple database	InterProScan	5.62-94.0
	Search against Uniprot Swissprot	BLAST+	2.14.1
	Search against KEGG ortholog database	BlastKOALA	3
	Prediction of transfer RNAs (tRNAs)	tRNAscan-SE	2.0.12
	Prediction of ribosomal RNAs (rRNAs)	RNAmmer	1.2
	GFF statistics, extract protein, etc.	AGAT	0.7.0
		gFACs	1.1.2
Miscellaneous	Dot-plot visualization	D-Genies (web)	n/a
	Collinearity calculation	McScanX	1.0*
	Collinearity visualization for synteny	Synvisio (web)	n/a
	Running docker images	Singularity	1.1.7-1.el9
	PacBio ccs reads	PacBio SMRT tools	10.1
	IsoSeq isoforms (full-length transcripts)	PacBio SMRT link	9.0

*Version of docker image used with singularity.

#### Draft *de novo* genome assembly

For general quality control of the input raw reads, we conducted a kmer analysis of the Illumina short read data (subsequently used for polishing). First, Kmergenie^25^ was used to estimate the appropriate kmer size for our sample, and then kmers were counted using Jellyfish^26^ to produce a kmer profile (histogram), which was then plotted by GenomeScope. We selected the GenomeScope^27^ pipeline for kmer profiling due to the capabilities of this software to provide overall genome characteristics from raw, short-read DNA sequencing data, without the need of a reference genome. From the resulting kmer profile produced by GenomeScope, the presence of repeats should be visible as pronounced peaks, while potential presence of sequencing errors and repeat duplicates would distort the appearance of the kmer histogram, due to increased variances and low frequency kmers^27^. The genome assembly pipeline (Fig. 1a) started with an initial draft assembly of PacBio long reads using Flye 2.9^28^, followed by a polishing step (error correction) using the tool POLCA from MaSurCa^29^, and short read data as input. Next, the Arima pipeline (https://github.com/ArimaGenomics/mapping_pipeline) was used to map Hi-C paired-end reads against the assembly, followed by the AllHiC pipeline for scaffolding of polyploid genomes^30^. We chose the AllHiC pipeline because it is specialized in avoiding that Hi-C signals erroneously link allelic haplotypes together in polyploids (or species with recent whole-genome duplications such as the crucian carp). With the scaffolded assembly, Juicebox^31^ was used to visualize Hi-C contact points, as well as to correct visibly misassembled scaffolds. BUSCO (Benchmarking Universal Single-Copy Orthologs^32^) scores were compared, before and after Juicebox curations, and with different levels of curation (minimum, medium, and high), to assess whether manual curation had an improving effect. QUAST^33^ was used to obtain length statistics of the draft genome at different steps of the assembly process. The final assembly was also checked with FCS-GX^34^ to detect potential contamination with genetic material from other organisms.

The structural annotation pipeline (described below) required filtering of the primary assembly. All contigs that were above 3000 bp were kept, plus one contig that was only 2959 bp long, but had more than 100 reads mapping (to investigate read support, RNA-seq data were mapped to the draft genome using STAR^35^, and the samtools^36^ command ‘idxstats’ was used to extract the number of reads aligning to each scaffold/contig). A total of 262 contigs were kept. After filtering, but before structural annotation was carried out, the assembled scaffolds were reordered by decreasing size and renamed using Funannotate (https://github.com/nextgenusfs/funannotate). Synteny between the largest scaffolds was visualized in Synvisio^37^, based on intra-genomic collinearity blocks calculated using McScanX^38^. This resulted in pairs that were renamed as their corresponding chromosome and sub-genome (A or B), with a total of 50 scaffolds (chromosomes), as expected from previous knowledge of the crucian carp and goldfish^39^. The remaining scaffolds were named with the prefix “scaffold”. The final subset genome assembly was soft-masked using RepeatModeler2 (https://www.repeatmasker.org/RepeatModeler/)^40^.

#### Structural annotation

The sequence representing the mitochondrial genome was identified using Blast+^41^ with an existing crucian carp mitochondrial genome^42,43^ as the query sequence and the *de novo* genome assembly as the target database. This search matched one scaffold (renamed ‘scaffold_107_mito’). The mitochondrial genes were annotated on the scaffold using MitoAnnotator from the MitoFish database^44–46^. Transfer RNAs were predicted using tRNAScan-SE^47^ while ribosomal RNAs were predicted using RNAmmer^48^ (Fig. 1b).

For the purpose of using RNA-seq data in the structural annotation of protein-coding genes (Fig. 1b), low-quality reads and adapters were trimmed from the libraries using Trimgalore (https://github.com/FelixKrueger/TrimGalore), whereafter read coverage was normalized using the Trinity pipeline^49^ script ‘insilico_read_normalization.pl’ (https://github.com/trinityrnaseq/trinityrnaseq/wiki/Trinity-Insilico-Normalization) with option ‘--max_cov 30’ to reduce the total number of reads included for annotation, while maximizing information across the genome, including regions with low expression. After coverage normalization, the reads (128.7 million pairs) were mapped to the filtered genome (262 scaffolds) using STAR^35^, with the following parameters: ‘--twopassMode Basic --outFilterMultimapNmax 1 --outSJfilterReads Unique --outSJfilterCountUniqueMin 6 3 3 3 --outSAMtype BAM SortedByCoordinate --outSAMstrandField intronMotif --outSAMattributes All’. By using only uniquely mapping reads and increasing the number of alignments needed for splice junctions to be included, we lowered the risk of including spurious gene models in the annotation. In the final alignment map (.bam) used for annotation, 120.8 million read pairs (93.84%) were uniquely mapped and properly paired.

For the final structural annotation of protein-coding genes (Fig. 1b), we first performed *ab initio* gene prediction with BRAKER3^50^. Training of the gene detection was performed with protein sequences from ray-finned fishes (OrthoDB v10 Actinopterygii dataset^51^) combined with proteins predicted from a genome of a farmed crucian carp from United Kingdom sequenced by the Wellcome Sanger Institute for the Darwin Tree of Life project^10^. The PASA pipeline^52^ was used to obtain an updated structural annotation that included annotation of untranslated regions (UTRs) and improved gene-isoform relationships. Exon and transcript lengths were obtained with gFACs^53^.

#### Functional annotation of protein-coding genes

For the functional annotation (Fig. 1c), we used AGAT (https://github.com/NBISweden/AGAT) to extract predicted transcript and protein sequences from the final assembly (using the final structural annotation), and then those proteins were searched for in the UniProtKB/Swiss-Prot database^54^ using Blast + and in the InterPro^55^ database using InterProScan^56^. The latter included searches against several databases focused on protein motifs. Gene ontology (GO) terms were extracted for genes based on the matching UniProtKB/Swiss-Prot protein entry. In addition, predicted proteins were searched for in the KEGG ortholog database using BlastKOALA^57^.

## Data Records

A list of input and final output data is given in Table 2, including relevant step in the pipeline (Fig. 1), name of the repository where data are available, type of data, and accession information or file name. All sequence data are deposited in the NCBI sequence read archive (SRA) under BioProject number PRJNA1119394^58^. The Whole Genome Shotgun project (i.e. the full genome assembly) has been deposited at GenBank under the accession JBEDAC000000000. The version described in this paper is version JBEDAC010000000^59^. The subset and soft-masked assemblies, together with structural and functional annotation files, as well as clustered high-quality transcript isoforms, are deposited in DataverseNO^60^. The files in DataverseNO are organised into six subfolders: 01_genome, 02_structural_annotation, 03_predicted_sequences, 04_functional_annotation, and 05_mitochondrial_genome_annotation.

**Table 2:** Data record details for input and output files.

Step	Archive	Description	Accession/File
1-in	SRA	PacBio SMRT Sequel II long DNA reads	SRR29316387
2-in	SRA	Illumina DNA reads	SRR29316385
3-in	SRA	Illumina Hi-C reads	SRR29316386
5-out	Genome	Assembly with 290 scaffolds	JBEDAC000000000
6-out	DvNO	Assembly after filtering to 262 scaffolds	01a_ccar_genome_v1_262scaffolds_fasta.txt
7-out	DvNO	Soft-masked version of subset genome	01b_ccar_genome_v1_262scaffolds_sm_fasta.txt
8a-out	DvNO	Transfer RNAs	02a_ccar_genome_v1_262scaffolds_trna_gff3.txt
8b-out	DvNO	Ribosomal RNAs	02b_ccar_genome_v1_262scaffolds_rrna_gff3.txt
9-in	SRA	Illumina RNA-seq reads from multiple tissues and individuals	SRR30720712
11-in	SRA	PacBio CCS reads from multiple tissues	SRR31178203
13a-in	DvNO	PacBio IsoSeq HQ isoforms	02c_ccar_isoseq_hq_transcripts_fasta.txt
13c-out	DvNO	Final structural annotation	02d_ccar_annotation_v5_gff3.txt
14-out	DvNO	Protein sequences	03a_ccar_annotation_v5_proteins_fasta.txt
		Transcript sequences	03b_ccar_annotation_v5_transcripts_fasta.txt
15a-out	DvNO	Kegg BlastKOALA output	04a_ccar_annotation_v5_kegg.txt
15b-out	DvNO	Interproscan output	04b_ccar_annotation_v5_interproscan.txt
15c-out	DvNO	Blast + output	04c_ccar_annotation_v5_swissprot_wGO_outfmt6.txt
15c-out	DvNO	Proteins and GO terms	04d_ccar_annotation_v5_swissprot_hits_and_GO_v2.txt
mito	DvNO	Mitochondrial genes	05_ccar_genome_v1_scaffold_107_mito_NCBI.txt

SRA, NCBI Sequence Read Archive; DvNO, DataverseNO; Genome, NCBI Genbank Genome. Data in NCBI SRA and Genome are deposited under BioProject number PRJNA1119394^58^. Data in DataverseNO are deposited under the handle GXMSUH^60^.

## Technical Validation

**Fig. 2:**
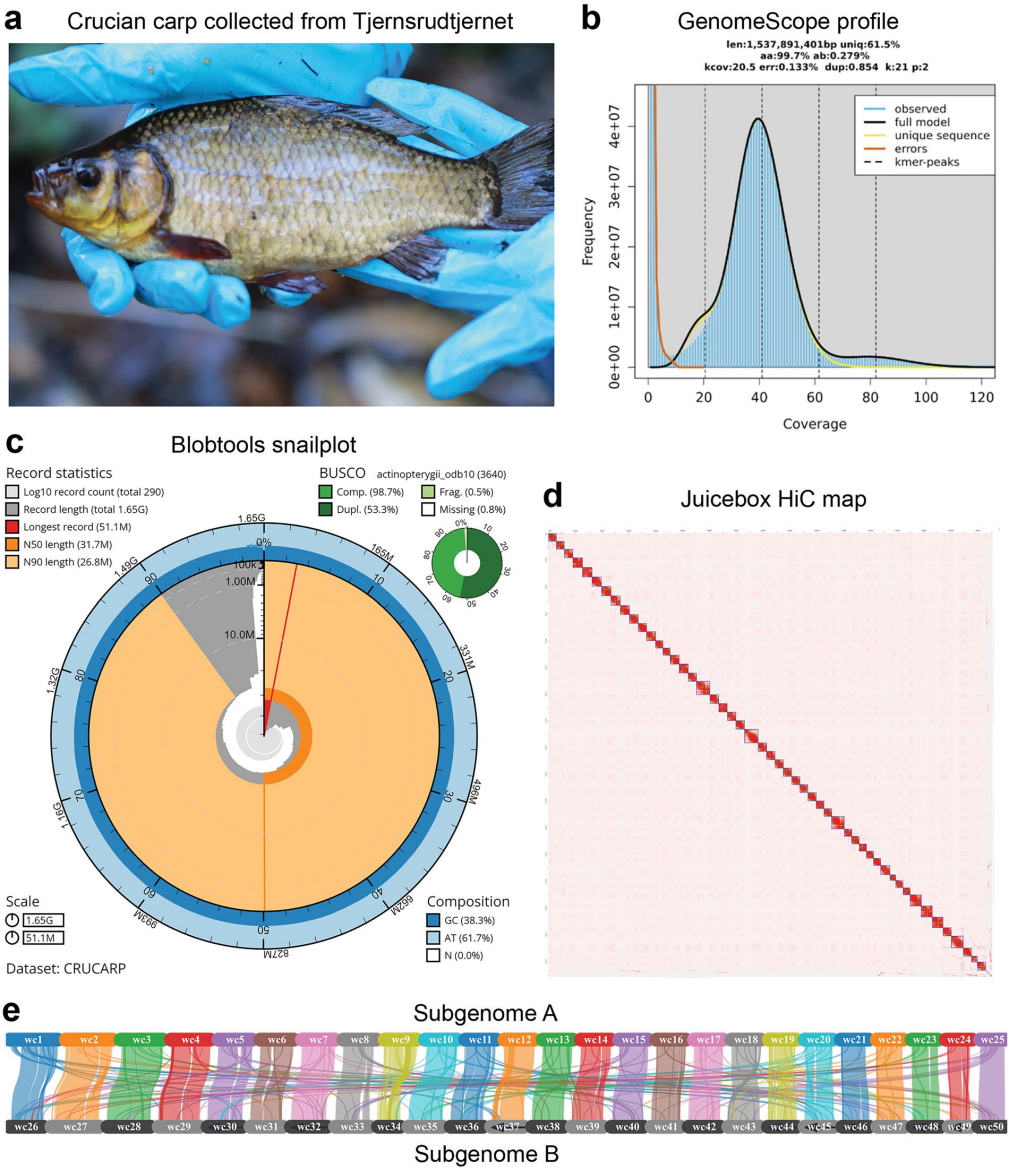
Genome of the wild crucian carp from Norway. (**a**) Wild crucian carp specimen collected in Tjernsrudtjernet pond (Oslo), by our research group during autumn. (**b**) Genomescope k-mer spectra that shows the fingerprint of a diploid without contamination. (**c**) Snail-plot visualization of the crucian carp assembly metrics. (**d**) Visualisation of chromatin contact points after mapping of Hi-C reads. After Juicebox curation, 50 scaffolds that were significantly larger than remaining scaffolds emerged, corresponding to the 50 chromosomes. (**e**) Collinearity analysis of the 50 scaffolds and synteny plotting reveals a pairing of the 50 scaffolds into two sub-genomes, which is expected in the crucian carp genome (collinearity blocks filtered with E value 1e-10 and minimum 7 genes). Note that in this figure, chromosomes named ccar-ua1 to ccar-ua25 in the assembly and annotation files are referred to as wc1 to wc25, while ccar-ub1 to ccar-ub25 are referred to as wc26-wc50 (due to requirements of MCScanX and Synvisio that were used for plotting).

We obtained a genome assembly from a wild-caught Norwegian crucian carp (Fig. 2a), with an estimated length of 1.65 Gbp, predicted by the GenomeScope k-mer plot based on short-read DNA data (Fig. 2b). The k-mer plot showed one main frequency peak at just below 40x coverage, indicating a high level of heterozygosity, with a much smaller secondary peak at 80x coverage. Furthermore, the k-mer plot indicated that most of the reads were included in the assembly. Assembly quality metrics are summarized in the Blobtools snail plot^61^ (Fig. 2c), and showed a high degree of completeness in terms of BUSCO. The longest scaffold of the genome was 51.1 Mbp (red line), while the shortest contig at 50% of the total assembly length (N50) was 31.7 Mbp (dark orange), and the shortest scaffold at 90% of the total assembly length (N90) was 26.8 Mbp (light orange). Among the 290 scaffolded contigs, after manual curation of the draft genome using Hi-C data, 50 scaffolds appeared that were markedly larger than the remaining scaffolds and covered 98.6% of the total length of the assembly. Specifically, when sorted by length the 50^th^ scaffold was 21 Mbp while the 51^st^ scaffold was 2.2 Mbp, and taken together the 50 largest scaffolds can therefore be assumed to correspond to the expected 50 chromosomes of the crucian carp (Fig. 2d). Based on the protein sequences predicted through functional annotation of the genome (see further below), blocks of collinearity could be identified, and showed the expected pairing of the 25 chromosome pairs reflecting the two sub-genomes (Fig. 2e), originating from the whole genome duplication specific to carps.

**Fig. 3:**
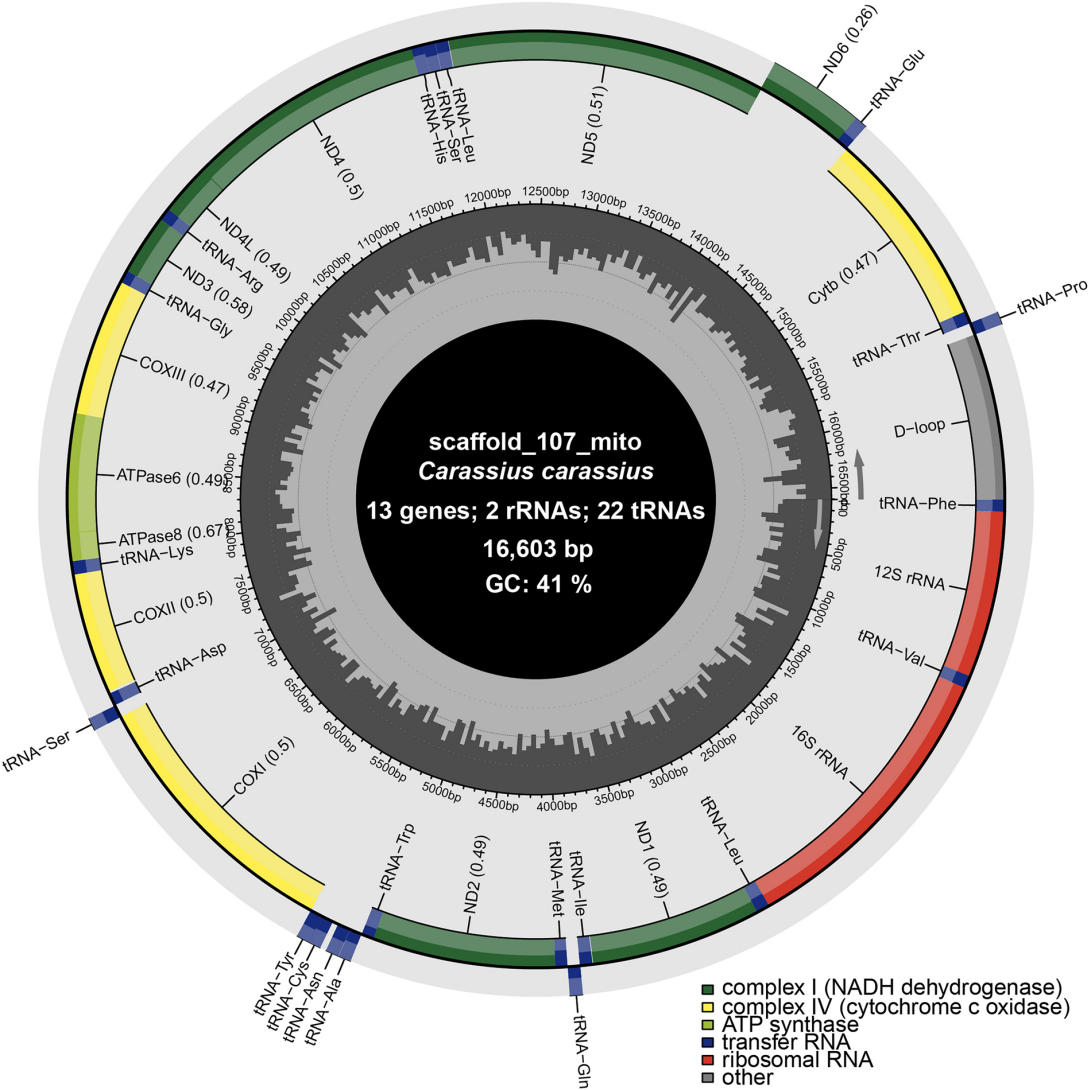
Crucian carp mitochondrial genome. The contig representing the mitochondrial genome was identified by running blastn (BLAST+) of an available crucian carp mitochondrial genome against a database of the sequences in the present genome assembly. Plot created with MitoFish.

In addition to the successful assembly of near-complete chromosomes, the mitochondrial genome was identified among the contigs assembled by Flye (i.e. one contig with no gaps). The length of the sequence (16 603 bp) was similar to the expected size of mitochondrial genomes, and the expected number and identity of protein-coding and non-coding genes were annotated (Fig. 3).

**Fig. 4:**
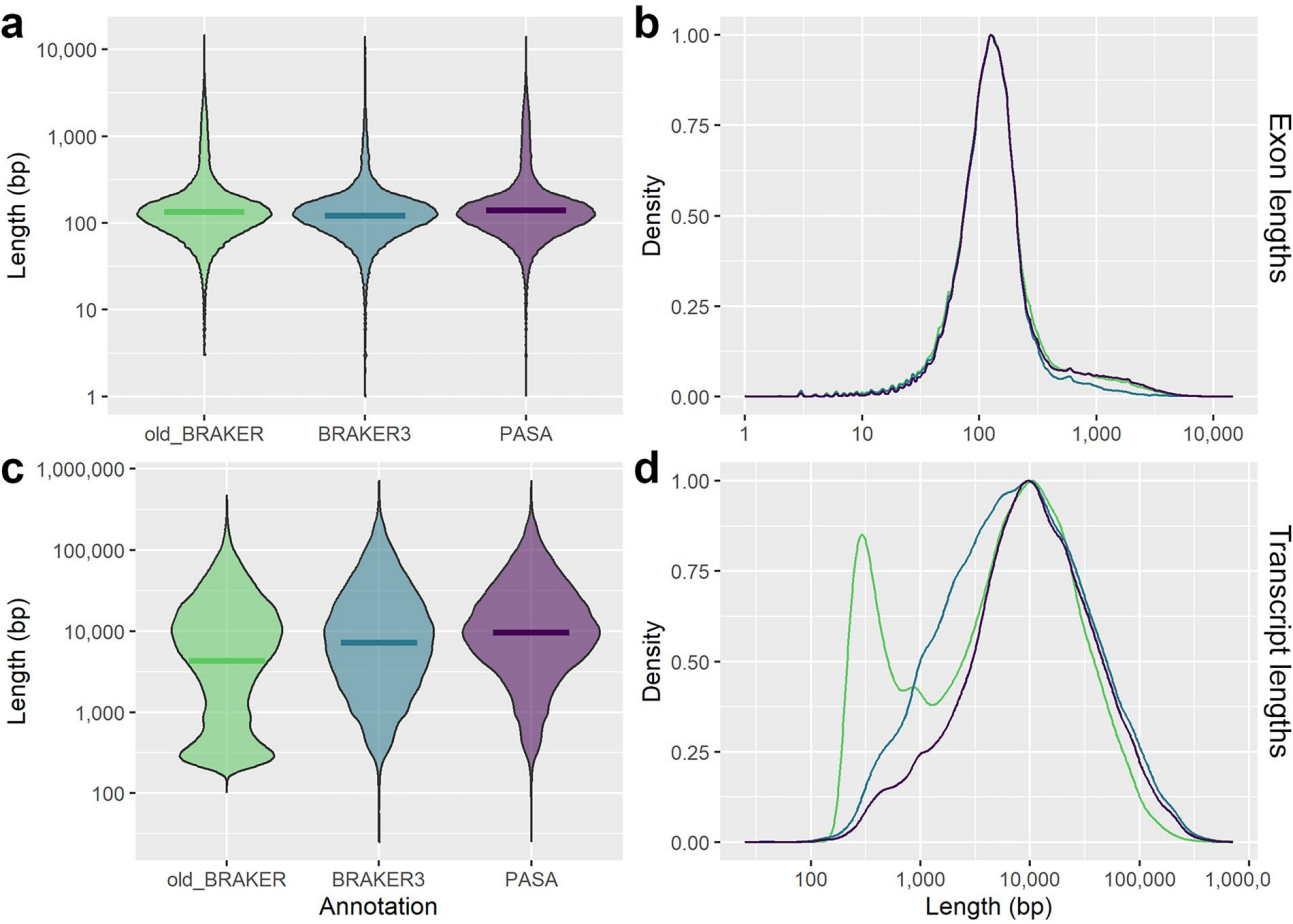
Exon and transcript lengths after structural annotation. The graphs show violin plots (**a,c**) and length density distributions (**b,d**) for exon lengths (**a,b**) and transcript lengths (**c,d**) after structural annotation using three different methods. The annotations being compared are ‘old_BRAKER’ in green (BRAKER1 and BRAKER2 merged by TSEBRA, and followed by refinement by PASA), ‘BRAKER3’ in blue (output from BRAKER3 pipeline alone), and ‘PASA’ (BRAKER3 followed by PASA).

The structural annotation pipeline using BRAKER3 and PASA resulted in a total of 82,557 protein-coding transcripts contained in 45,667 genes (Table 3). The number of transcripts went up from 63,098 before the PASA step, indicating that the PASA pipeline using IsoSeq full-length transcripts helped significantly to resolve gene-isoform relationships and likely also recovered splice variants not detected, or discarded, by BRAKER3. We also compared the final structural annotation with an earlier version obtained using the previous version of BRAKER (consisting of running BRAKER1^62^ and BRAKER2^63^ separately, then merging them with TSEBRA^64^, followed by PASA). This earlier approach resulted in a larger total number of genes, of which a large proportion were mono-exonic (Table 3). Also the exon length (Fig. 4a,b) and total transcript length (Fig. 4c,d) were improved with the final annotation, compared to both BRAKER3 alone and the previous version of BRAKER. The most notable effect of refining transcripts using PASA was on the length of multi-exonic transcripts, which almost doubled, likely due to the addition of UTRs and inclusion of some exons previously annotated as separate, mono-exonic genes. Overall, the PASA annotation was considered a worthwhile improvement of the annotation quality.

**Table 3:** Annotation metrics.

Attribute	‘old_BRAKER’	BRAKER3	BRAKER3 + PASA
Number genes^a^	79,060	45,771	45,667
Number transcripts	129,667	63,098	82,557
Number multi-exonic genes	50,080	41,872	42,523
Number mono-exonic genes	28,980	3,899	3,144
Number of 5′-UTRs	38,389	1	44,666
Number of 3′-UTRs	39,491	1	45,265
Mean multi-exonic CDS size (bp)	4,770	2,807	5,023
Median multi-exonic CDS size (bp)	2,344	1,410	2,585
Median number exons^b^	10	8	10
Unique IPR terms detected			14,189
Genes with Interpro (IPR) term			43,617
Genes with Swissprot match and GO term(s)			41,373
Genes with KEGG orthologs (KO)			29,798

^a^only protein coding. In addition, 6 394 transfer RNAs (tRNA) and 4 551 ribosomal RNAs (rRNA) were annotated. ^b^per multi-exonic gene.

**Fig. 5:**
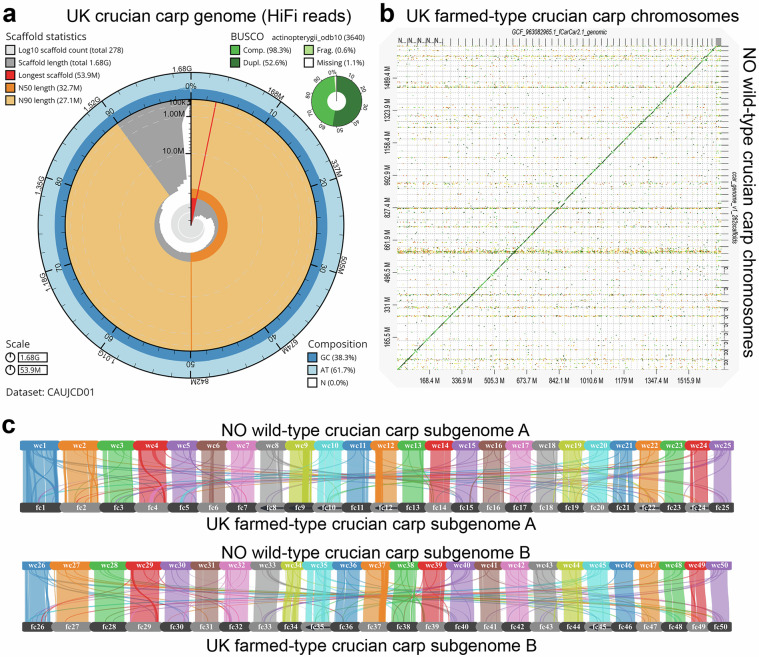
Comparison of genome assemblies from farmed (UK) crucian carp genome and wild (NO) crucian carp. (**a**) Blobtools snailplot summary of the farmed crucian carp genome^10^. This assembly was built using PacBio HiFi reads, and it shows that the genome we have obtained using PacBio long read sequencing with short-read error correction has a similarly high quality (shown in Fig. 2c). (**b**) Dgenies dotplot of the 50 chromosomes of farmed crucian carp compared to the crucian carp genome presented in this study. The plot indicates, as expected, high degree of similarity and continuity but also some chromosomes with possible structural differences. (**c**) Synvisio synteny plot (collinearity blocks filtered with e value 1e-10 and minimum 7 genes) of similarity between the crucian carp from the present study (chromosome names wc01 to wc25 for subgenome A, and wc26 to wc50 for subgenome B), against the farmed crucian carp (chromosome names fc01 to fc25 for subgenome A, and fc26 to fc50 for subgenome B).

Recently, a chromosome-level genome assembly generated using PacBio HiFi data from a farmed crucian carp from the UK was released by the Darwin Tree-of-Life initiative (https://portal.darwintreeoflife.org/)^10^, and was therefore compared in more detail to the genome assembly of the present study. Snailplots (Fig. 2c vs. 5a) indicated that scaffold-level length metrics were only marginally better for the HiFi assembly. A dot-plot made with Dgenies^65^ (Fig. 5b) revealed high levels of sequence identity, an equal number of chromosomes, and similar sizes of scaffolds between the assemblies. Collinearity analysis^38^ and visualization of synteny between the assemblies^37^ (Fig. 5c) also showed the expected pairing of chromosomes within the two sub-genomes. These comparisons also indicate that there could be some structural differences between the assemblies (e.g. translocations), which is expected due to the variation that exists between the methods used to obtain sequencing data and the assembly pipelines, but also the likely biological differences between the source populations of the specimens, where the wild-type crucian carp population is known to be exposed to seasonal anoxia, which is unlikely to be the case for the farmed crucian carp. While the assemblies were similar in many aspects, the contiguity of our assembly, however, was substantially better when compared across a number of different contig-level metrics (Table 4): the contig level N50 was 15Mbp for our CLR-Flye assembly, compared to 3.8Mbp for the HiFi-Hifiasm assembly, and the contig L50 in our genome was 40, while it was 135 for the HiFi-Hifiasm assembly. A better contiguity may explain why the present assembly, despite the slightly shorter total length and scaffold-level N50, still obtained a higher BUSCO score and annotated a larger number of protein-coding genes (Table 4).

**Table 4:** Assembly metrics with comparison to other cyprinid genomes.

Attribute	*C. carassius* (w)	*C. carassius* (f)	*C. gibelio*	*C. auratus*	*Cyprinus carpio*
Sequencing	74x PacBio CLR Illumina HiSeq, HiC	43x PacBio HiFi, Arima2 Hi-C	45x PacBio HiFi, OmniC Illumina reads	71x PacBio CLR	185x PacBio CLR, Oxford Nanopore, Illumina HiSeq
Assembly	Flye, MaSuRCA-POLCA, AllHiC, Arima pipeline, Juicebox	Hifiasm, purge_dups, YaHS	Hifiasm unitigger, 3D-dna	Canu	wtdbg, quickmerge
	MitoAnnotator	MitoHiFi	n/a	cloning	cloning
Total length^a^	1,654,898	1,684,296	1,583,352	1,820,629	1,671,603
Ungapped length	1,654,776	1,684,071	1,582,055	1,820,404	1,663519
GC content (%)	38.27	38.31	37.63	37.48	37.09
BUSCOs (%)^b^:	99.6	99.3	99.5	99.3	98.6
Protein-coding genes	45,667	41,837	43,901	53,065	43,531
Chromosomes	50	50	50	59	50
Scaffolds	261^c^	238	51^c^	6,213	1,975
Scaffold N50	31,733	32,664	30,678	22,763	29,545
Scaffold N90	26,847	27,139	26,107	87	20,764
Scaffold auN	33,279	33,770	33,145	18,403	29,423
Scaffold L50	23	23	22	32	24
Scaffold L90	45	45	44	1,508	49
Ns per 100 kbp	4	10	82	12	474
Longest scaffold	51,097	53,934	55,533	37,185	48,440
Contigs	941	1,070	2,648	8,460	14,642
Contig N50	15,168	3,822	5,134	821	1,574
Contig N90	2,094	1,005	885	74	121
Contig auN	14,386	4,570	6,406	1,399	2,876
Contig L50	40	135	91	513	226
Contig L90	149	462	343	3,543	1,764

^a^All lengths in kbp. ^b^These BUSCO scores for complete orthologs are either calculated as part of the BRAKER3 pipeline (wild-type crucian carp, *C*. *carassius* (w)) or sourced from NCBI genome information (all others). They differ slightly from scores obtained in Blobtool snail plot (Figs. 2c, 5a), which are likely estimated from the genome rather than the predicted proteins. ^c^Including the scaffold identified as MT.

**Fig. 6:**
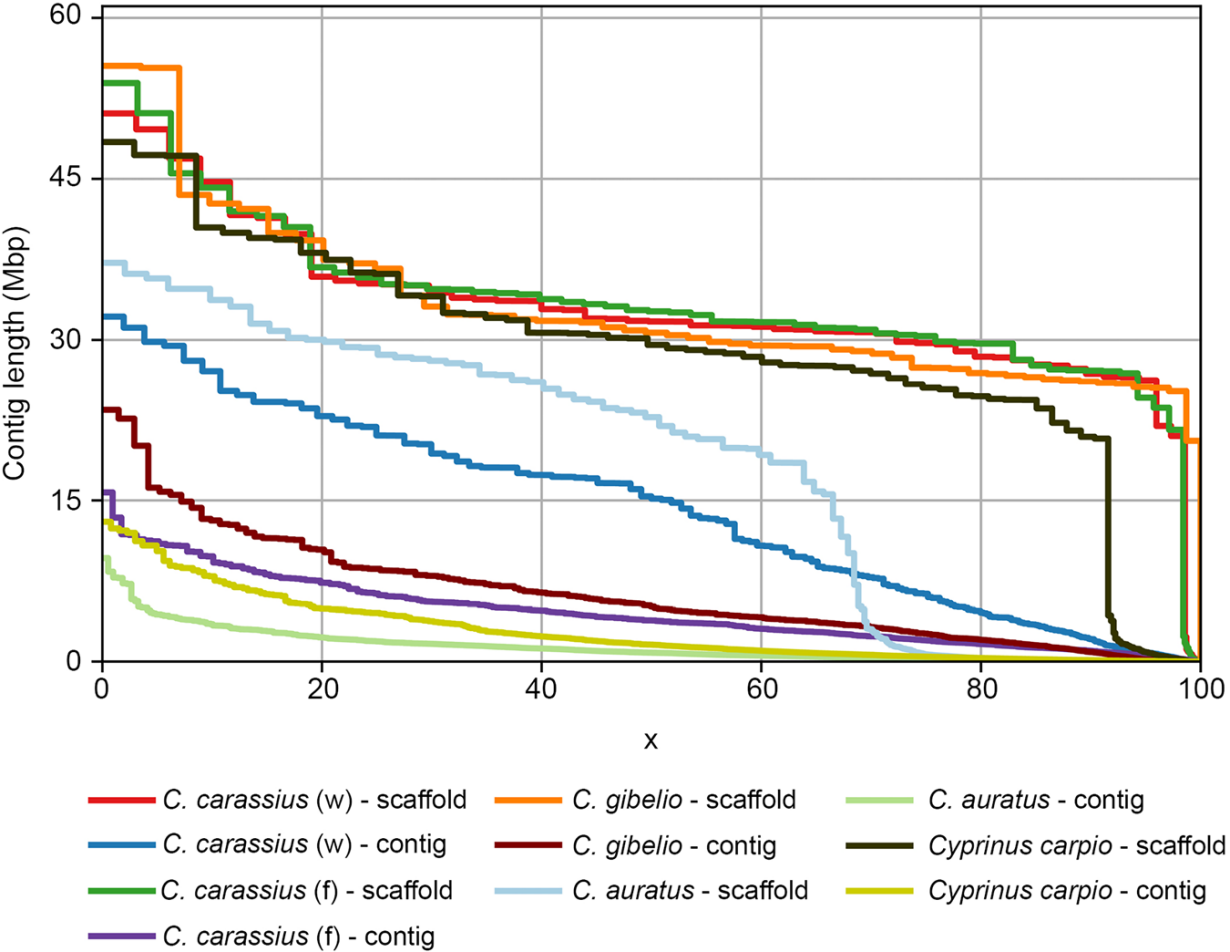
Cumulative length of contigs (Nx). Data are shown for the scaffolded assemblies as well as for the ‘broken’ assemblies (contig level). Plot generated using QUAST optimised for large genomes with option “--large”. *C. carassius* (w) is the genome presented in this paper.

Chromosome-level genome assemblies are also available for the related species goldfish, silver crucian carp and common carp^5,6,66^, and the present genome of crucian carp is similar in terms of overall size, number of chromosomes and GC content to these genomes, but importantly is much less fragmented (Table 4), with a contig level N50 of 15 Mbp, which is 3- to 18-fold longer than the other assemblies. This is particularly visible when inspecting the cumulative length of contigs (Fig. 6). Here, it can be seen that the wild-type, farmed and silver crucian carp genomes perform best at the scaffold level, with common carp following closely behind. At the contig level, the present crucian carp genome is markedly above the other assemblies. It is also noted that the reference genome for goldfish (i.e. labelled as reference genome in NCBI and available at ensemble.org) appears to be of lower quality and has 59 chromosomes, which is not the expected number, based on the evolutionary history and relatedness to common carp and crucian carp, that both have the expected 50 (twice as many as zebrafish, *Danio rerio*). Furthermore, while the reference genome for goldfish appears to be longer (1.8 Gb) than both crucian and common carp, the scaffold L90 is very large, and not closer to the number of chromosomes as is the case for the common carp and both crucian carp genomes.

Taken together, these results show that our sequencing efforts have resulted in a high-quality chromosome-level reference genome for the wild-type crucian carp. Considering the additional data used for structural annotation, specifically the full-length transcripts from multiple tissues and mRNA sequencing from both a variety of tissues and anoxia treatments, we are confident that our genome assembly is representative of the wild, anoxia-tolerant crucian carp and represents a significant resource for future studies regarding the evolution of mechanisms involved in anoxia survival.

## Data Availability

No customised scripts or coding were utilized in this study. For all analyses, the software package and versions are shown in Table 1 under Methods. Unless otherwise stated, default parameters were used.

## References

[1] Nilsson, G. E. & Lutz, P. L. Anoxia Tolerant Brains. *J. Cereb. Blood Flow Metab.* **24**, 475–486, 10.1097/00004647-200405000-00001 (2004). 15129179 10.1097/00004647-200405000-00001

[2] Lefevre, S. & Nilsson, G. E. Two decades of research on anoxia tolerance – mitochondria, -omics and physiological diversity. *J. Exp. Biol.* **226**, jeb245584, 10.1242/jeb.245584 (2023). 37042631 10.1242/jeb.245584

[3] Lefevre, S. & Nilsson, G. E. Case study: The anoxia-tolerant crucian carp. In *Encyclopedia of Fish Physiology (Second Edition)* (eds. Alderman, S. L. & Gillis, T. E.) 148–158. 10.1016/B978-0-323-90801-6.00105-1 (Academic Press, Oxford, 2024).

[4] Fagernes, C. E. *et al*. Extreme anoxia tolerance in crucian carp and goldfish through neofunctionalization of duplicated genes creating a new ethanol-producing pyruvate decarboxylase pathway. *Sci. Rep.* **7**, 7884, 10.1038/s41598-017-07385-4 (2017). 28801642 10.1038/s41598-017-07385-4 PMC5554223

[5] Chen, Z. *et al*. De novo assembly of the goldfish (*Carassius auratus*) genome and the evolution of genes after whole-genome duplication. *Sci. Adv.* **5**, eaav0547, 10.1126/sciadv.aav0547 (2019). 31249862 10.1126/sciadv.aav0547 PMC6594761

[6] Xu, P. *et al*. The allotetraploid origin and asymmetrical genome evolution of the common carp *Cyprinus carpio*. *Nat. Commun.* **10**, 4625, 10.1038/s41467-019-12644-1 (2019). 31604932 10.1038/s41467-019-12644-1 PMC6789147

[7] Li, J.-T. *et al*. Parallel subgenome structure and divergent expression evolution of allo-tetraploid common carp and goldfish. *Nat. Genet.* **53**, 1493–1503, 10.1038/s41588-021-00933-9 (2021). 34594040 10.1038/s41588-021-00933-9 PMC8492472

[8] Van den Thillart, G., Van Berge-Henegouwen, M. & Kesbeke, F. Anaerobic metabolism of goldfish, *Carassius auratus* (L.): Ethanol and CO_2_ excretion rates and anoxia tolerance at 20, 10 and 5 °C. *Comp. Biochem. Physiol. Part A Physiol.* **76**, 295–300, 10.1016/0300-9629(83)90330-4 (1983).

[9] Zhou, B. S. *et al*. Metabolic adjustments in the common carp during prolonged hypoxia. *J. Fish Biol.* **57**, 1160–1171, 10.1111/j.1095-8649.2000.tb00478.x (2000).

[10] *NCBI Genbank* https://identifiers.org/ncbi/insdc.gca:GCA_963082965.1 (2023).

[11] *NCBI Genbank* https://identifiers.org/ncbi/insdc.gca:GCA_023724105.1 (2021).

[12] *NCBI Genbank* https://identifiers.org/ncbi/insdc.gca:GCA_003368295.1 (2018).

[13] *NCBI Genbank* https://identifiers.org/ncbi/insdc.gca:GCA_018340385.1 (2021).

[14] Lefevre, S. *et al*. Re-oxygenation after anoxia induces brain cell death and memory loss in the anoxia-tolerant crucian carp. *J. Exp. Biol.* **220**, 3883–3895, 10.1242/jeb.165118 (2017). 29093186 10.1242/jeb.165118

[15] Gerber, L. *et al*. Expression of Prolyl Hydroxylase Domains (PHDs), the upstream regulator of HIF, in the Brain of the Anoxia-Tolerant Crucian Carp during Anoxia-Reoxygenation. *Am. J. Physiol. Integr. Comp. Physiol*. **326**, R184–R195. 10.1152/ajpregu.00211.2023. 10.1152/ajpregu.00211.2023 38145292

[16] Gerber, L., Torp, M.-K., Nilsson, G. E., Lefevre, S. & Stensløkken, K.-O. Differential production of mitochondrial reactive oxygen species between mouse (*Mus musculus*) and crucian carp (*Carassius carassius*). *Acta Physiol.* **240**, e14244, 10.1111/apha.14244 (2024). 10.1111/apha.14244 39463199

[17] Dahl, H.-A., Johansen, A., Nilsson, G. E. & Lefevre, S. The Metabolomic Response of Crucian Carp (*Carassius carassius*) to Anoxia and Reoxygenation Differs between Tissues and Hints at Uncharacterized Survival Strategies. *Metabolites* **11**, 435, 10.3390/metabo11070435 (2021). 34357329 10.3390/metabo11070435 PMC8304758

[18] Riggs, C. L. *et al*. Small Non-coding RNA Expression and Vertebrate Anoxia Tolerance. *Front. Genet.* **9**, 230, 10.3389/fgene.2018.00230 (2018). 30042786 10.3389/fgene.2018.00230 PMC6048248

[19] Scott, M. A., Fagernes, C. E., Nilsson, G. E. & Stensløkken, K.-O. Maintained mitochondrial integrity without oxygen in the anoxia-tolerant crucian carp. *J. Exp. Biol.* **227**, jeb247409, 10.1242/jeb.247409 (2024). 38779846 10.1242/jeb.247409 PMC11418198

[20] Vornanen, M., Stecyk, J. A. W. & Nilsson, G. E. The Anoxia-Tolerant Crucian Carp (*Carassius carassius* L.). In *Hypoxia* (ed. Jeffrey, G. R.) **vol. 27** 397–441 10.1016/S1546-5098(08)00009-5 (Academic Press, 2009).

[21] Stecyk, J. A. W., Stensløkken, K.-O., Farrell, A. P. & Nilsson, G. E. Maintained Cardiac Pumping in Anoxic Crucian Carp. *Science* **306**, 77, 10.1126/science.1100763 (2004). 15459381 10.1126/science.1100763

[22] Johansen, A., Thiede, B., Anonsen, J. H. & Nilsson, G. E. Surviving without oxygen involves major tissue specific changes in the proteome of crucian carp (*Carassius carassius*). *PeerJ* **11**, e14890, 10.7717/peerj.14890 (2023). 36915662 10.7717/peerj.14890 PMC10007964

[23] Johansen, A., Thiede, B., Anonsen, J. H. & Nilsson, G. E. Phosphoproteomic changes in response to anoxia are tissue-specific in the anoxia-tolerant crucian carp (*Carassius carassius*). *Front. Physiol.* **15**, 1407834, 10.3389/fphys.2024.1407834 (2024). 38872833 10.3389/fphys.2024.1407834 PMC11170284

[24] de Meo, I., Østbye, K., Kahilainen, K. K. & Poléo, A. B. S. The role of predation risk in structuring life-history traits of crucian carp (*Carassius carassius*) in a series of small boreal lakes. *J. Fish Biol.* **103**, 939–949, 10.1111/jfb.15485 (2023). 37395556 10.1111/jfb.15485

[25] Chikhi, R. & Medvedev, P. Informed and automated k-mer size selection for genome assembly. *Bioinformatics* **30**, 31–37, 10.1093/bioinformatics/btt310 (2014). 23732276 10.1093/bioinformatics/btt310

[26] Marçais, G. & Kingsford, C. A fast, lock-free approach for efficient parallel counting of occurrences of k-mers. *Bioinformatics* **27**, 764–770, 10.1093/bioinformatics/btr011 (2011). 21217122 10.1093/bioinformatics/btr011 PMC3051319

[27] Vurture, G. W. *et al*. GenomeScope: fast reference-free genome profiling from short reads. *Bioinformatics* **33**, 2202–2204, 10.1093/bioinformatics/btx153 (2017). 28369201 10.1093/bioinformatics/btx153 PMC5870704

[28] Kolmogorov, M., Yuan, J., Lin, Y. & Pevzner, P. A. Assembly of long, error-prone reads using repeat graphs. *Nat. Biotechnol.* **37**, 540–546, 10.1038/s41587-019-0072-8 (2019). 30936562 10.1038/s41587-019-0072-8

[29] Zimin, A. V. & Salzberg, S. L. The genome polishing tool POLCA makes fast and accurate corrections in genome assemblies. *PLOS Comput. Biol.* **16**, e1007981, 10.1371/journal.pcbi.1007981 (2020). 32589667 10.1371/journal.pcbi.1007981 PMC7347232

[30] Zhang, X., Zhang, S., Zhao, Q., Ming, R. & Tang, H. Assembly of allele-aware, chromosomal-scale autopolyploid genomes based on Hi-C data. *Nat. Plants* **5**, 833–845, 10.1038/s41477-019-0487-8 (2019). 31383970 10.1038/s41477-019-0487-8

[31] Durand, N. C. *et al*. Juicebox Provides a Visualization System for Hi-C Contact Maps with Unlimited Zoom. *Cell Syst.* **3**, 99–101, 10.1016/j.cels.2015.07.012 (2016). 27467250 10.1016/j.cels.2015.07.012 PMC5596920

[32] Manni, M., Berkeley, M. R., Seppey, M., Simão, F. A. & Zdobnov, E. M. BUSCO Update: Novel and Streamlined Workflows along with Broader and Deeper Phylogenetic Coverage for Scoring of Eukaryotic, Prokaryotic, and Viral Genomes. *Mol. Biol. Evol.* **38**, 4647–4654, 10.1093/molbev/msab199 (2021). 34320186 10.1093/molbev/msab199 PMC8476166

[33] Gurevich, A., Saveliev, V., Vyahhi, N. & Tesler, G. QUAST: quality assessment tool for genome assemblies. *Bioinformatics* **29**, 1072–1075, 10.1093/bioinformatics/btt086 (2013). 23422339 10.1093/bioinformatics/btt086 PMC3624806

[34] Astashyn, A. *et al*. Rapid and sensitive detection of genome contamination at scale with FCS-GX. *Genome Biol* **25**, 60, 10.1186/s13059-024-03198-7 (2024). 38409096 10.1186/s13059-024-03198-7 PMC10898089

[35] Dobin, A. *et al*. STAR: ultrafast universal RNA-seq aligner. *Bioinformatics* **29**, 15–21, 10.1093/bioinformatics/bts635 (2013). 23104886 10.1093/bioinformatics/bts635 PMC3530905

[36] Li, H. *et al*. The Sequence Alignment/Map format and SAMtools. *Bioinformatics* **25**, 2078–2079, 10.1093/bioinformatics/btp352 (2009). 19505943 10.1093/bioinformatics/btp352 PMC2723002

[37] Bandi, V. & Gutwin, C. Interactive Exploration of Genomic Conservation. In *Proceedings of Graphics Interface 2020* 74–83. 10.20380/GI2020.09 (Canadian Human-Computer Communications Society/Société canadienne du dialogue humain-machine, 2020).

[38] Wang, Y. *et al*. MCScanX: a toolkit for detection and evolutionary analysis of gene synteny and collinearity. *Nucleic Acids Res.* **40**, e49–e49, 10.1093/nar/gkr1293 (2012). 22217600 10.1093/nar/gkr1293 PMC3326336

[39] Wang, Y. *et al*. Comparative genome anatomy reveals evolutionary insights into a unique amphitriploid fish. *Nat. Ecol. Evol.* **6**, 1354–1366, 10.1038/s41559-022-01813-z (2022). 35817827 10.1038/s41559-022-01813-z PMC9439954

[40] Flynn, J. M. *et al*. RepeatModeler2 for automated genomic discovery of transposable element families. *Proc. Natl. Acad. Sci.* **117**, 9451–9457, 10.1073/pnas.1921046117 (2020). 32300014 10.1073/pnas.1921046117 PMC7196820

[41] Camacho, C. *et al*. BLAST+: architecture and applications. *BMC Bioinformatics* **10**, 421, 10.1186/1471-2105-10-421 (2009). 20003500 10.1186/1471-2105-10-421 PMC2803857

[42] Guo, X., Liu, S. & Liu, Y. Evidence for maternal inheritance of mitochondrial DNA in allotetraploid. *DNA Seq.* **18**, 247–256, 10.1080/10425170701248541 (2007). 17541829 10.1080/10425170701248541

[43] Guo, X., Liu, S. & Liu, Y. *Carassius carassius* mitochondrion, complete genome. *NCBI GenBank* https://identifiers.org/nucleotide:NC_006291.1 (2004).

[44] Zhu, T., Sato, Y., Sado, T., Miya, M. & Iwasaki, W. MitoFish, MitoAnnotator, and MiFish Pipeline: Updates in 10 Years. *Mol. Biol. Evol.* **40**, msad035, 10.1093/molbev/msad035 (2023). 36857197 10.1093/molbev/msad035 PMC9989731

[45] Sato, Y., Miya, M., Fukunaga, T., Sado, T. & Iwasaki, W. MitoFish and MiFish Pipeline: A Mitochondrial Genome Database of Fish with an Analysis Pipeline for Environmental DNA Metabarcoding. *Mol. Biol. Evol.* **35**, 1553–1555, 10.1093/molbev/msy074 (2018). 29668970 10.1093/molbev/msy074 PMC5967551

[46] Iwasaki, W. *et al*. MitoFish and MitoAnnotator: a mitochondrial genome database of fish with an accurate and automatic annotation pipeline. *Mol. Biol. Evol.* **30**, 2531–2540, 10.1093/molbev/mst141 (2013). 23955518 10.1093/molbev/mst141 PMC3808866

[47] Chan, P. P., Lin, B. Y., Mak, A. J. & Lowe, T. M. tRNAscan-SE 2.0: improved detection and functional classification of transfer RNA genes. *Nucleic Acids Research* **49**, 9077–9096, 10.1093/nar/gkab688 (2021). 10.1093/nar/gkab688 PMC8450103 34417604

[48] Lagesen, K. *et al*. RNAmmer: consistent and rapid annotation of ribosomal RNA genes. *Nucleic Acids Res.* **35**, 3100–3108, 10.1093/nar/gkm160 (2007). 17452365 10.1093/nar/gkm160 PMC1888812

[49] Grabherr, M. G. *et al*. Full-length transcriptome assembly from RNA-Seq data without a reference genome. *Nat Biotechnol* **29**, 10.1038/nbt.1883(2011). 10.1038/nbt.1883 PMC3571712 21572440

[50] Gabriel, L. *et al*. BRAKER3: Fully automated genome annotation using RNA-seq and protein evidence with GeneMark-ETP, AUGUSTUS, and TSEBRA. *Genome Res.* **34**, 769–777, 10.1101/gr.278090.123 (2024). 38866550 10.1101/gr.278090.123 PMC11216308

[51] Kriventseva, E. V. *et al*. OrthoDB v10: sampling the diversity of animal, plant, fungal, protist, bacterial and viral genomes for evolutionary and functional annotations of orthologs. *Nucleic Acids Res.* **47**, D807–D811, 10.1093/nar/gky1053 (2019). 30395283 10.1093/nar/gky1053 PMC6323947

[52] Haas, B. J. *et al*. Automated eukaryotic gene structure annotation using EVidenceModeler and the Program to Assemble Spliced Alignments. *Genome Biol.* **9**, R7, 10.1186/gb-2008-9-1-r7 (2008). 18190707 10.1186/gb-2008-9-1-r7 PMC2395244

[53] Caballero, M. & Wegrzyn, J. gFACs: Gene Filtering, Analysis, and Conversion to Unify Genome Annotations Across Alignment and Gene Prediction Frameworks. *Genom. Proteom. Bioinform.* **17**, 305–310, 10.1016/j.gpb.2019.04.002 (2019). 10.1016/j.gpb.2019.04.002 PMC6818179 31437583

[54] The Uniprot Consortium. UniProt: the Universal Protein Knowledgebase in 2023. *Nucleic Acids Res.* **51**, D523–D531, 10.1093/nar/gkac1052 (2023). 36408920 10.1093/nar/gkac1052 PMC9825514

[55] Blum, M. *et al*. The InterPro protein families and domains database: 20 years on. *Nucleic Acids Res.* **49**, D344–D354, 10.1093/nar/gkaa977 (2021). 33156333 10.1093/nar/gkaa977 PMC7778928

[56] Jones, P. *et al*. InterProScan 5: genome-scale protein function classification. *Bioinformatics* **30**, 1236–1240, 10.1093/bioinformatics/btu031 (2014). 24451626 10.1093/bioinformatics/btu031 PMC3998142

[57] Kanehisa, M., Sato, Y. & Morishima, K. BlastKOALA and GhostKOALA: KEGG Tools for Functional Characterization of Genome and Metagenome Sequences. *J. Mol. Biol.* **428**, 726–731, 10.1016/j.jmb.2015.11.006 (2016). 26585406 10.1016/j.jmb.2015.11.006

[58] *NCBI Sequence Read Archive*, https://identifiers.org/ncbi/insdc.sra:SRP512373 (2025).

[59] *NCBI Genbank*, https://identifiers.org/ncbi/insdc.gca:GCA_047456465.1 (2025).

[60] Valencia-Pesqueira, L. M., Jentoft, S., Hoff, S. N. K., Tørresen, O. K. & Lefevre, S. Replication Data for: Chromosome-level de novo genome assembly of wild, anoxia-tolerant crucian carp. *Carassius carassius.*10.18710/GXMSUH (2024).10.1038/s41597-025-04813-340128231

[61] Challis, R., Richards, E., Rajan, J., Cochrane, G. & Blaxter, M. BlobToolKit - Interactive Quality Assessment of Genome Assemblies. *G3 Genes|Genomes|Genetics* **10**, 1361–1374, 10.1534/g3.119.400908 (2020). 32071071 10.1534/g3.119.400908 PMC7144090

[62] Hoff, K. J., Lange, S., Lomsadze, A., Borodovsky, M. & Stanke, M. BRAKER1: Unsupervised RNA-Seq-Based Genome Annotation with GeneMark-ET and AUGUSTUS. *Bioinformatics* **32**, 767–769, 10.1093/bioinformatics/btv661 (2016). 26559507 10.1093/bioinformatics/btv661 PMC6078167

[63] Brůna, T., Hoff, K. J., Lomsadze, A., Stanke, M. & Borodovsky, M. BRAKER2: automatic eukaryotic genome annotation with GeneMark-EP+ and AUGUSTUS supported by a protein database. *NAR Genomics Bioinforma.* **3**, lqaa108, 10.1093/nargab/lqaa108 (2021). 10.1093/nargab/lqaa108 PMC7787252 33575650

[64] Gabriel, L., Hoff, K. J., Brůna, T., Borodovsky, M. & Stanke, M. TSEBRA: transcript selector for BRAKER. *BMC Bioinformatics* **22**, 566, 10.1186/s12859-021-04482-0 (2021). 34823473 10.1186/s12859-021-04482-0 PMC8620231

[65] Cabanettes, F. & Klopp, C. D-GENIES: dot plot large genomes in an interactive, efficient and simple way. *PeerJ* **6**, e4958, 10.7717/peerj.4958 (2018). 29888139 10.7717/peerj.4958 PMC5991294

[66] Kuhl, H. *et al*. Equilibrated evolution of the mixed auto-/allopolyploid haplotype-resolved genome of the invasive hexaploid Prussian carp. *Nat. Comm.* **13**, 4092, 10.1038/s41467-022-31515-w (2022). 10.1038/s41467-022-31515-w PMC9283417 35835759

